# Optimizing treatment strategies for pediatric phimosis and redundant prepuce: a comparative study of traditional circumcision and disposable circumcision stapler

**DOI:** 10.3389/fped.2024.1394403

**Published:** 2024-07-22

**Authors:** Guoyan Zhang, Yongliang Luo, Shangchao Cheng, Yonglang Tu, Xiangyu Meng, Linde Wu, Gang Li, Xiyuan Chen

**Affiliations:** ^1^ Physical examination and Rehabilitation Department, Kunming City Maternal and Child Health Hospital, Kunming, Yunnan, China; ^2^ Surgery, Yunnan Maternal and Child Health Hospital, Kunming, Yunnan, China; ^3^ Surgery, The First Affiliated Hospital of Changzhi Medical College, Changzhi, Shanxi, China; ^4^ Surgery, The First Affiliated Hospital of Kunming Medical University, Kunming, Yunnan, China; ^5^ Surgery, Honghe Autonomous Prefecture 3rd Hospital, Gejiu, Yunnan, China

**Keywords:** pediatric, phimosis, redundant prepuce, circumcision, disposable circumcision stapler

## Abstract

**Objective:**

To investigate the surgical outcomes and complication rates of traditional circumcision and disposable circumcision stapler in the treatment of pediatric patients with phimosis and redundant prepuce.

**Methods:**

A retrospective analysis was conducted on pediatric patients with phimosis or preputial redundancy treated at our pediatric surgery department from January 2022 to December 2023. The patients were divided into two groups: treated with traditional circumcision (control group) and treated with a disposable circumcision stapler (experimental group). Surgical parameters (operation time, intraoperative bleeding), postoperative outcomes (postoperative pain scores, wound healing time, severe edge swelling, wound dehiscence, postoperative rebleeding, postoperative infection, aesthetic satisfaction), were compared between the two groups.

**Results:**

A total of 301 pediatric patients were included in our study, with 146 in the traditional group and 155 in the stapler group. The stapler group showed significantly lower values in operation time, intraoperative bleeding, and postoperative rebleeding compared to the traditional group (*P* < 0.05). However, the traditional group had a significant advantage in postoperative wound healing time and the occurrence of severe edge swelling (*P* < 0.05). There were no significant differences between the two groups in terms of anesthetic drug dosage, postoperative pain level, postoperative infection rate, wound dehiscence, and aesthetic satisfaction (*P* > 0.05).

**Conclusion:**

In the treatment of pediatric phimosis and redundant prepuce, the advantage of traditional circumcision lies in faster postoperative recovery and less severe edge swelling. The disposable circumcision stapler excels in thorough hemostasis, easy and safe operation, suitable for primary medical use, but lags behind in postoperative recovery compared to the traditional method. Each treatment approach has its own advantages, and the choice should be based on the actual condition and circumstances of the patient. Personalized treatment decisions should be made collaboratively to achieve the best therapeutic outcomes.

## 1 Introduction

**Figure 1:**
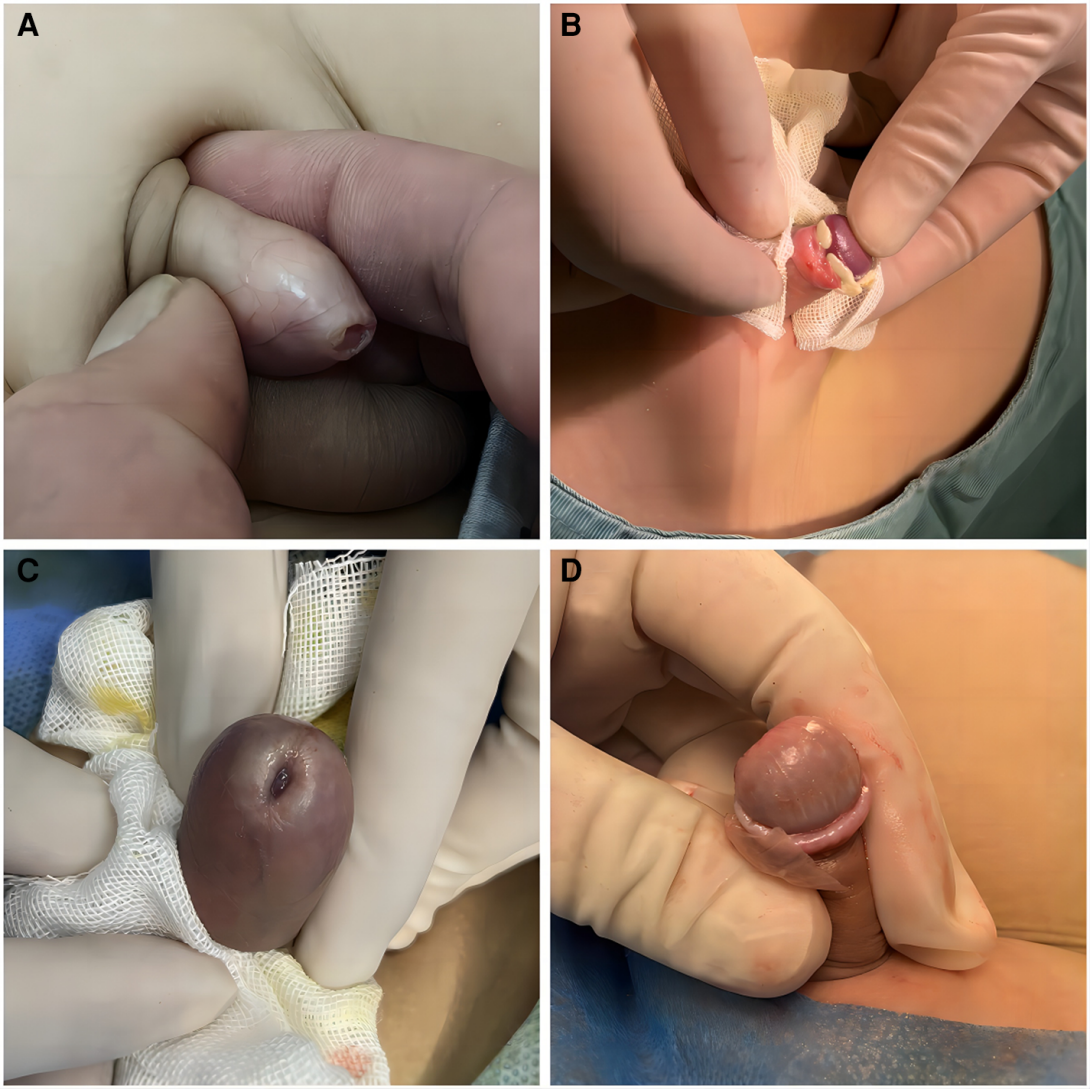
(**A**) This is a case of phimosis, characterized by a narrowed preputial opening that restricts the full retraction of the foreskin, resulting in the inability to expose the glans penis. (**B**) Smegma detected during surgery, which is a white lumps formed by sloughed epithelial cells under the foreskin, located around the corona. It may lead to complications such as local infection, adhesions, balanitis, and Balanitis xerotica obliterans (BXO). (**C**) BXO is an acquired, non-contagious, chronic inflammatory skin disease that typically affects the foreskin, glans penis, and prepuce, resulting in sclerotic scarring, secondary phimosis, urinary difficulties, sexual dysfunction, and even potentially malignant transformations. (**D**) The most common symptoms of BXO are foreskin scars and sclerosis, there may also be evidence of pallor and similar thickening of the glans surface.

Redundant prepuce and phimosis are common and frequently occurring conditions in children (1, 2). Due to the accumulation of secretions, urine, and other substances under the prepuce, prolonged exposure can lead to local infections, adhesions, smegma, balanitis, Balanitis xerotica obliterans (BXO), and other complications (Figure 1), with severe cases causing significant long-term psychological and physiological effects on affected individuals, especially children (3, 4). Treatment approaches for pediatric phimosis and redundant prepuce vary across different countries and ethnicities (5). Studies indicate that early intervention significantly improves the prognosis of phimosis (4, 6). Currently, surgical circumcision is the primary method for treating phimosis and redundant prepuce in children (7). For children with surgical indications, prepuce circumcision is the main surgical approach (8), reducing the occurrence of local infections, smegma, BXO, and related complications (9). Additionally, it has a certain effect in preventing HIV/AIDS and penile cancer (10).

In recent years, various male circumcision techniques have emerged, including traditional methods, sleeve prepuce circumcision, Ali's clamping method, and disposable circumcision stapler (11, 12). Each technique carries different risks and advantages in pediatric surgery, with traditional circumcision and the removal of auxiliary materials dominating the field (13). Previous research indicates that traditional prepuce circumcision is associated with longer operation times, surgical scars, relatively complex procedures, and uneven surgical margins (14). In contrast, the removal of auxiliary materials, particularly the successful application of disposable circumcision staplers, offers advantages such as faster surgery, no need for suture closure, neat margins, minimal bleeding, and shorter duration (15). This approach has gained high praise from scholars, showing widespread application and achieving good clinical efficacy (13, 16). The purpose of this study was to compare the clinical outcomes of traditional prepuce circumcision and disposable circumcision stapler in the treatment of pediatric phimosis and redundant prepuce. The details of the study are reported below.

## 2 Materials and methods

### 2.1 Preoperative evaluation

All patients were in good general health, with a history of recurrent urinary tract infections, BXO, or other surgical indications. Routine blood tests, urinalysis, and coagulation function tests were normal. Preoperative evaluations ruled out chronic infections and urinary tract infections. The surgeries, either traditional method (Group A, control group) or disposable circumcision stapler (Group B, experimental group), were performed under local anesthesia. Inclusion criteria were a diagnosis of phimosis or redundant prepuce, corresponding surgical indications, and informed consent for participation in the study. Exclusion criteria were abnormal preoperative examinations, coagulation disorders, severe liver or kidney diseases, short frenulum, concealed penis, hypospadias, and other surgical contraindications. The study received ethical approval from the hospital's ethics committee (YNFY2022-13), and all patients provided informed consent.

### 2.2 Research methods

Both surgeries were conducted under local anesthesia, performed by the same group of physicians, and preceded by preoperative examinations including routine blood tests, coagulation function tests, blood-borne infectious diseases, and urinalysis. The anesthesia method consisted of dorsal nerve block anesthesia and infiltrative root block anesthesia (17), using 0.5% lidocaine injection (Tianjin Jinyao Pharmaceutical Co., Ltd., China, China National Medical Products Administration Approval Number: H12021000). The dosage, adjusted based on pain intensity, did not exceed 4 mg/kg (18).

#### 2.2.1 Group A

Traditional Surgical Approach Patients were placed in a supine position (12, 13). After routine disinfection and draping, local anesthesia with dorsal nerve block anesthesia and infiltrative root block anesthesia was administered. Two hemostatic forceps were clamped on the dorsal and ventral sides of the prepuce, spaced approximately 0.5 cm apart. Using tissue scissors, adhesions were separated, and the excess prepuce was circumferentially excised at a position 0.5–0.8 cm from the coronal sulcus, leaving 0.1–0.2 cm longer length at the ligature site compared to the dorsal prepuce. Hemostasis was achieved through electrocoagulation and ligation. Interrupted suturing with 4-0 absorbable surgical thread was used for aligning and suturing the inner and outer layers of the prepuce. Elastic bandages were applied for fixation. Dressings were changed on the second postoperative day, followed by dressing changes every 2–3 days until wound healing, with suture removal on the 7th postoperative day.

#### 2.2.2 Group B

**Figure 2:**
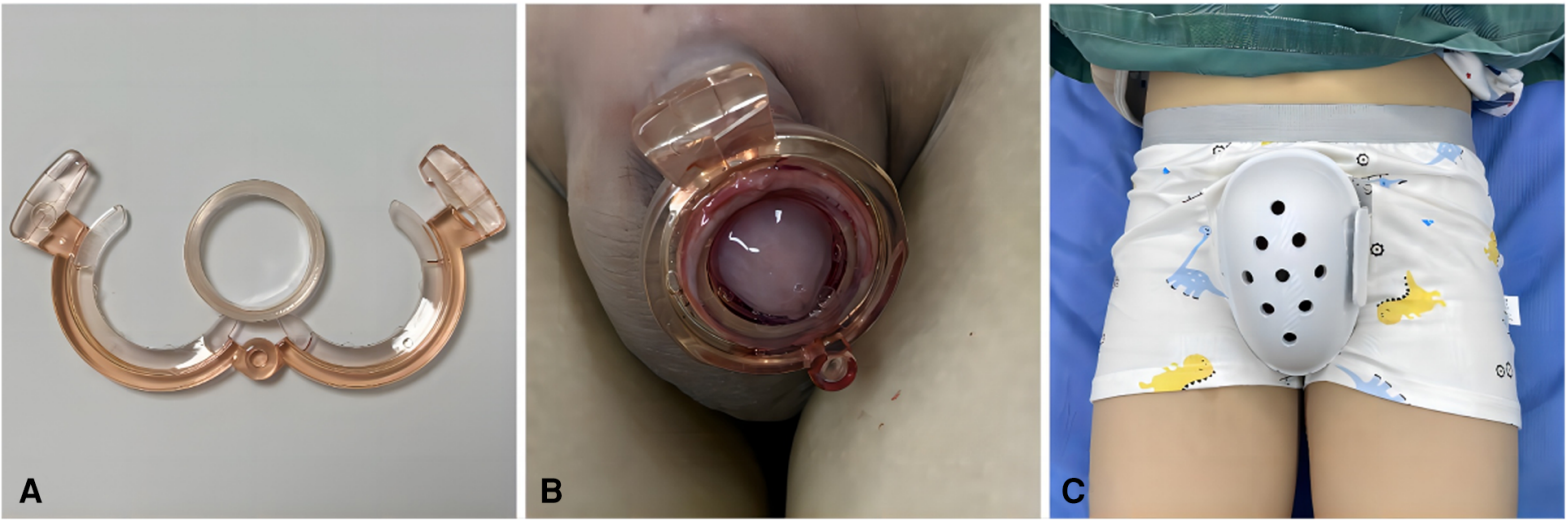
(**A**) The Chinese Shang Ring, comprising an inner ring and an outer ring, is applied by inserting the inner ring into the inner aspect of the foreskin and advancing it to the coronal sulcus. After adjusting the position of the foreskin, the outer ring is assembled and the two locking teeth are secured, thereby completing the surgical procedure. (**B**) Postoperative status of the Shang Ring: Following surgery, the inner and outer rings of the Shang Ring are securely in place, with redundant foreskin trimmed and the glans penis exposed. (**C**) “Protective shorts”: These are designed specifically for postoperative protection of the pediatric perineal region, aiming to prevent accidental contact or injury to the surgical site during movement or unforeseen situations.

Disposable Circumcision Stapler (“Shang Ring”) (19). The Shang Ring, designed and manufactured by Anhui Wuhu Shengda Medical Instrument Technology Co., Ltd., with registration certificate number: Wanxie Registration 20172020095, and model specifications of A-Z type, was used. Patients were placed in a supine position with the penis in a non-erect state. The appropriate size of the stapler was determined using a special measurement ruler. After routine disinfection and draping, local anesthesia was administered as described before. For patients with a narrow preputial orifice, a mosquito forceps was used for dilation. The dorsal prepuce was incised and inverted for patients with adhesions, and smegma was removed. The stapler's inner ring was inserted and advanced to the coronal sulcus. Symmetrical forceps were clamped at the 12 o'clock, 6 o'clock, 3 o'clock, and 9 o'clock positions on the outer edge of the prepuce, lifting it up. The prepuce was everted, positioned onto the inner ring, and the inner and outer layers of the prepuce were adjusted to the appropriate position. The outer ring was then fitted, leaving approximately 0.8–1.0 cm of the inner layer. After accurate engagement of the outer ring into the inner ring, the first fixing tooth was secured. The inner layer was readjusted to the desired position, and the second tooth was secured. Excess prepuce was excised. Postoperatively, the stapler was checked, and it was removed 10–14 days after the procedure based on the situation. As shown in Figure 2.

#### 2.2.3 Postoperative management

(1) Avoid strenuous activities and heavy physical labor, and rest quietly for 24–48 h. (2) Change the dressing once on the day after the surgery, followed by dressing changes every 2–3 days until wound healing or ring removal, to prevent surgical site infections. (3) Depending on individual circumstances, apply “lidocaine cream” topically or administer “ibuprofen” orally for pain relief. (4) Instruct patients not to withhold urine, reduce water intake at night to prevent urinary retention leading to penile erection pain, bleeding, etc. (5) Monitor postoperative conditions; in case of persistent bleeding, incisional dehiscence, excessive swelling, or other complications, prompt hospital return for treatment is necessary.

#### 2.2.4 Evaluation criteria

Compare relevant surgical indicators between the two groups, including intraoperative parameters such as surgical time, intraoperative bleeding volume, and anesthetic drug dosage (20). Postoperative complications evaluated comprise pain intensity, incision healing time, severe edge swelling, postoperative rebleeding rate, incision dehiscence, postoperative infection, and satisfaction with appearance (14). Surgical time is calculated from the start of anesthesia to the end of surgery. Intraoperative bleeding volume is measured using a 5 cm × 5 cm gauze, with 5 ml of bleeding calculated. Postoperative pain intensity is assessed using the visual analogue scale (VAS) ranging from 0 to 10 points (21). Edge severe swelling is defined as swelling diameter exceeding 30% of the normal penile diameter (16). Postoperative rebleeding refers to cases where active bleeding persists after local compression for 5 min. Satisfaction with appearance is subjectively evaluated by patients or their guardians during a 1-month follow-up, categorized as satisfied or dissatisfied (22).

### 2.3 Statistical analysis

Statistical analysis of research data was performed using SPSS 22.0 statistical software. Descriptive statistics are presented as x ± s for continuous data. Group comparisons were conducted using *t*-tests and χ^2^ tests for continuous and categorical data, respectively. A significance level of *P* < 0.05 was considered statistically significant.

## 3 Results

### 3.1 Clinical data

We collected data from 301 pediatric patients with redundant prepuce or phimosis treated at our pediatric surgery department from January 2022 to December 2023, with 146 in the traditional group and 155 in the stapler group. There were no statistically significant differences in general patient characteristics (ethnic group, age, weight, height) between the two groups (*P* > 0.05). The total treatment costs between the two patient groups, Group A incurred a cost of 248.98 ± 10.55 (USD), while Group B, due to the use of disposable circumcision stapler, had a cost of 264.39 ± 12.67 (USD), This difference was statistically significant, with a *P* < 0.001. As shown in Table 1.

**Table 1:** Comparison of demographic, clinical characteristics, and treatment costs between two groups.

Variable	Group A	Group B	*P*-value
Ethnic group	Han Nationality	Han Nationality	–
Residence	Kunming City	Kunming City	–
Family medical history	Neg	Neg	–
Age (years)	9.91 ± 3.25	10.13 ± 3.14	0.551
Weight (kg)	39.82 ± 6.02	41.02 ± 5.36	0.068
Height (cm)	130.58 ± 9.34	132.03 ± 10.59	0.210
Phimosis	55	57	0.834
Urinary tract infection	49	53	0.847
Balanitis xerotica obliterans	5	7	0.649
Treatment costs (USD)	248.98 ± 10.55	264.39 ± 12.67	<0.001

### 3.2 Clinical outcomes

Group A exhibited superior outcomes in terms of incision healing time and the occurrence rate of severe edge swelling compared to Group B, with *P* < 0.05 (Tables 2, 3). In the comparison of surgical time, intraoperative bleeding, and postoperative rebleeding, Group B showed significant advantages over Group A, with *P* < 0.05 (Tables 2, 3).

**Table 2:** Comparative analysis of surgical indicators (operating time, blood loss, incision healing time, and anesthetic dose) between two groups ($\bar{x}\pm s$).

Index	Group A	Group B	*t*-value	*P*-value
Operating time (min)	21.09 ± 3.20	9.87 ± 1.66	18.692	<0.001
Blood loss (ml)	9.85 ± 1.54	3.92 ± 1.03	18.745	<0.001
Incision healing time (days)	8.36 ± 2.52	14.15 ± 5.36	−5.368	<0.001
Anesthetic dose (ml)	4.26 ± 1.07	4.39 ± 1.18	0.458	0.646

**Table 3:** Comparison of postoperative outcomes and patient satisfaction between two groups [*n* (%)].

Index	Group A	Group B	*χ* ^2^	*P*-value
Severe edge swelling [*n* (%)]	15 (10.27)	39 (25.16)	7.937	0.005
Postoperative rebleeding [*n* (%)]	9 (6.16)	2 (1.29)	4.711	0.030
Postoperative infection [*n* (%)]	9 (6.16)	8 (5.16)	0.127	0.722
Wound dehiscence [*n* (%)]	6 (4.10)	7 (4.52)	0.028	0.868
Aesthetic satisfaction [*n* (%)]	129 (88.36)	131 (84.52)	0.069	0.793

The unit of all indicators is the number of person times, and the percentage is in brackets.

**Figure 3:**
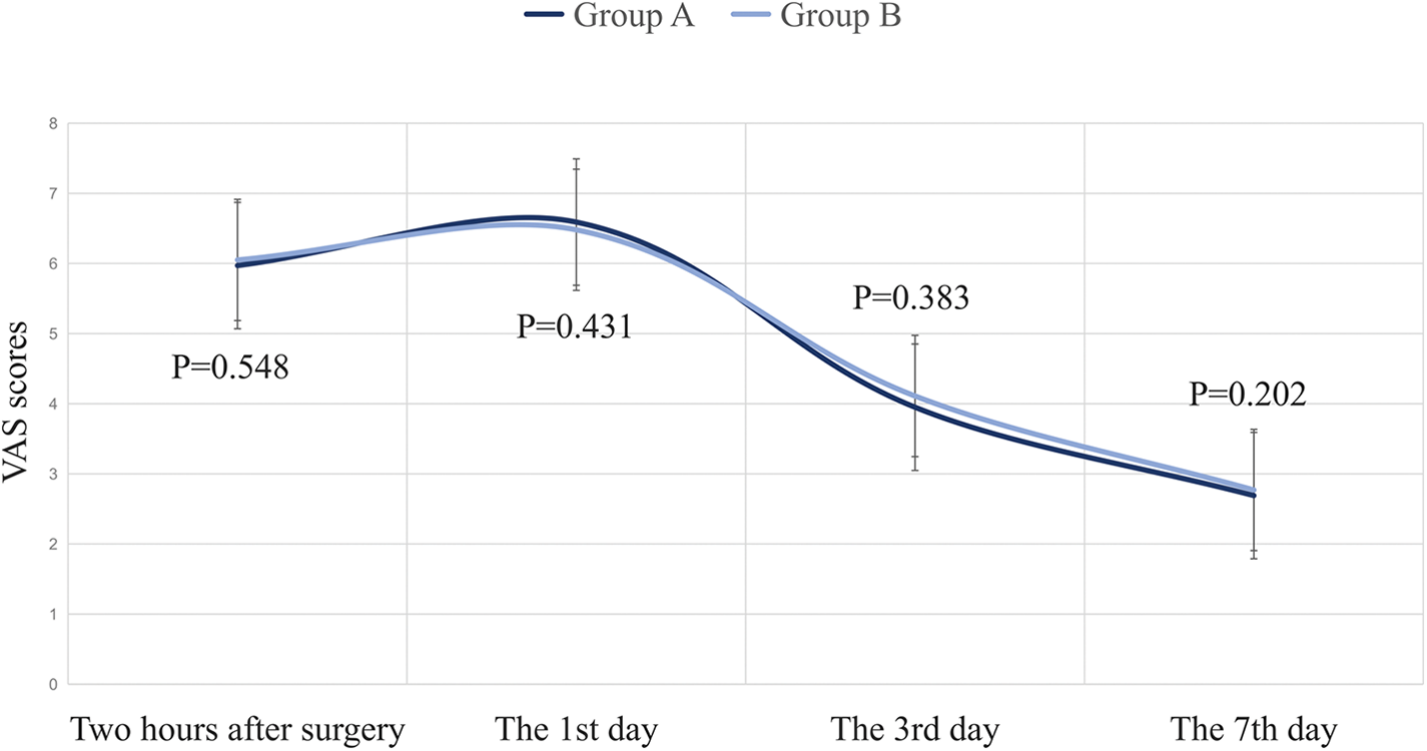
Postoperative pain scores in two groups (x ± s). The horizontal axis represents the postoperative follow-up time. The vertical axis represents the degree of postoperative pain evaluated using Visual Analog Scale (VAS), with a score range of 0–10 points. At 0 points, it indicates complete pain, while at 10 points, it indicates complete intolerance of pain. There was no significant difference (*P* > 0.05) in the comparison of data between the two groups at two hours, 1 day, 3 days, and 7 days after surgery.

In terms of anesthetic drug dosage, postoperative infection, incision dehiscence, and satisfaction with appearance, there was no significant difference between the two surgical methods, with *P* > 0.05 (Tables 2, 3). Regarding postoperative pain intensity, it peaked from 2 h postoperatively to the first day postoperatively, but there was no significant difference in the comparison between the two groups, with *P* > 0.05 (Figure 3). No severe complications occurred in any cases, and there were no patients requiring additional surgeries.

## 4 Discussion

Phimosis and redundant prepuce in pediatric patients remain common conditions (1), the main method for treating is circumcision, which is mainly divided into traditional surgery and resection surgery using disposable auxiliary materials (13). The ideal circumcision procedure should encompass attributes such as safety, simplicity, rapid recovery, minimal pain, high satisfaction with appearance, fewer complications, and minimal impact on daily life (23). The choice between traditional prepuce circumcision and disposable circumcision stapler surgeries involves weighing various factors (24, 25). Our study, comparing these two approaches, yielded insights into their respective advantages and drawbacks.

This study results indicate that the group using disposable circumcision staplers excelled in operative time, intraoperative bleeding, and postoperative bleeding compared to the traditional group. However, the traditional group demonstrated certain advantages in terms of incision healing time and edge swelling. These findings align with prior research. Regarding postoperative pain, both groups exhibited peak pain levels at 2 h and on the first day postoperatively, with no significant differences between the two groups at different time points. This suggests no significant disparity in postoperative pain experiences for patients undergoing either type of surgery (26). Postoperative pain is a crucial concern influencing the choice of surgical method, making pain management a vital aspect of future research (27). Both surgical methods demonstrated favorable outcomes in terms of postoperative reinfection, incision dehiscence, and satisfaction with appearance. Ultimately, traditional circumcision surgeries tend to have longer operative times and face relatively higher bleeding risks, but they offer quicker postoperative recovery, less severe prepuce swelling, and lower costs. On the other hand, disposable circumcision stapler surgeries are safe and straightforward, ensuring thorough hemostasis, but they have a longer treatment duration and higher costs. Thus, both methods have their advantages and disadvantages.

In our study, there were 5 patients in Group A and 7 patients in Group B diagnosed with BXO. Due to long-term and repeated inflammatory stimulation, BXO leads to atrophy, adhesion, scarring, and hardening of the foreskin at the urethral orifice, resulting in brittle texture and easy bleeding. Pathologically, it is characterized by chronic inflammation of the glans penis and foreskin, tissue thickening and hardening, lymphocyte infiltration, atrophy, and adhesion (3). After surgical intervention in both groups, we observed that Group B exhibited significantly better operating time outcomes compared to Group A, *P* < 0.001. However, there were no statistically significant differences in other complications such as blood loss, wound healing time, and anesthetic dosage (*P* > 0.05). It is hypothesized that the local adhesion and brittle texture caused by BXO may contribute to increased blood loss and prolonged incision healing time, in Table 4. Given the limited number of cases, further observations and studies with a larger sample size are warranted.

**Table 4:** Comparative analysis of surgical indicators (operating time, blood loss, incision healing time, and anesthetic dose) between two groups of patients with Balanitis Xerotica Obliterans (BXO) ($\bar{x}\pm s$).

Index	Group A (*n* = 5)	Group B (*n* = 7)	*t*-value	*P*-value
Operating time (min)	27.09 ± 4.31	12.50 ± 2.92	7.035	<0.001
Blood loss (ml)	15.03 ± 2.87	12.90 ± 2.35	1.415	0.187
Incision healing time (days)	15.42 ± 3.81	16.33 ± 4.01	−0.395	0.701
Anesthetic dose (ml)	6.39 ± 1.70	5.92 ± 1.56	0.496	0.630

In addition, one limitation of our study is the relatively short follow-up period. Extended follow-up is essential for evaluating the durability of results, potential late complications, and the overall impact of selected surgical methods on patients' quality of life. Future studies should consider prolonging the follow-up period for a more comprehensive understanding of the postoperative process. Additionally, this study did not explicitly delve into potential outcome differences based on racial or cultural variations (28). These factors may influence the prevalence, presentation, and treatment management of phimosis (29). Subsequent studies should explore the impact of race and culture on the condition and its surgical treatment.

In light of the current emphasis on informed consent in the medical environment (30), it is crucial to accurately present the risks and benefits associated with various surgical procedures to guardians and patients (31). The professionals should comprehensively consider factors such as the patient's age, medical condition, local healthcare standards, surgical environment, and postoperative care when selecting a surgical approach. This holistic approach aims to enhance surgical outcomes and patient satisfaction. Simultaneously, we aspire to furnish physicians with more evidence-based guidance for choosing appropriate surgical methods and to offer insights for future research directions.

## Data Availability

The original contributions presented in the study are included in the article/Supplementary Material, further inquiries can be directed to the corresponding author.
